# Advancements in non-grape fruit wine production: A review of recent progress, challenges, and prospects

**DOI:** 10.1016/j.fochx.2026.104249

**Published:** 2026-07-26

**Authors:** Xiao Gong, Ningli Qi, Biaoshi Wang, Tinghui Chen, Yang Luo, Yiming Luo

**Affiliations:** a College of food science and engineering, Lingnan Normal University, Zhanjiang 524048, China; b Laboratory of Quality & Safety Risk Assessment on Agro-Products Processing (Zhanjiang), Ministry of Agriculture and Rural Affairs, Agricultural Products Processing Research Institute, Chinese Academy of Tropical Agricultural Sciences, Zhanjiang 524091, China; c College of Notoginseng Medicine and Pharmacy, Wenshan University, Wenshan 663099, China; d College of Food Science and Technology, Huazhong Agricultural University, Wuhan 430070, China

**Keywords:** Non-grape fruit wine, Inherent limitations, Fermentation, Haze, Browning, Safety

## Abstract

Non-grape fruits offer diverse nutritional and health-promoting properties; however, substantial post-harvest losses persist due to inadequate handling and storage infrastructure. Fruit wine fermentation represents a promising strategy for valorizing surplus and substandard fruit, thereby mitigating waste while enhancing economic value. Nevertheless, current production protocols largely draw upon grape winemaking experience, constraining the differentiated development of non-grape fruit wine enterprises. Unlike grape wine, the quality and safety of non-grape fruit wines hinge on precise compensation for raw material deficiencies, targeted regulation of fermentation parameters, and a deliberate equilibrium between stability and sensory quality. This review systematically summarizes critical challenges encountered across the practical fruit wine production chain, encompassing juice pre-treatment, fermentation technologies, and post-fermentation stability, while outlining future research directions. It aims to provide guidance for process innovation and product standardization in the fruit wine industry.

## 1 Introduction

**Fig. 1:**
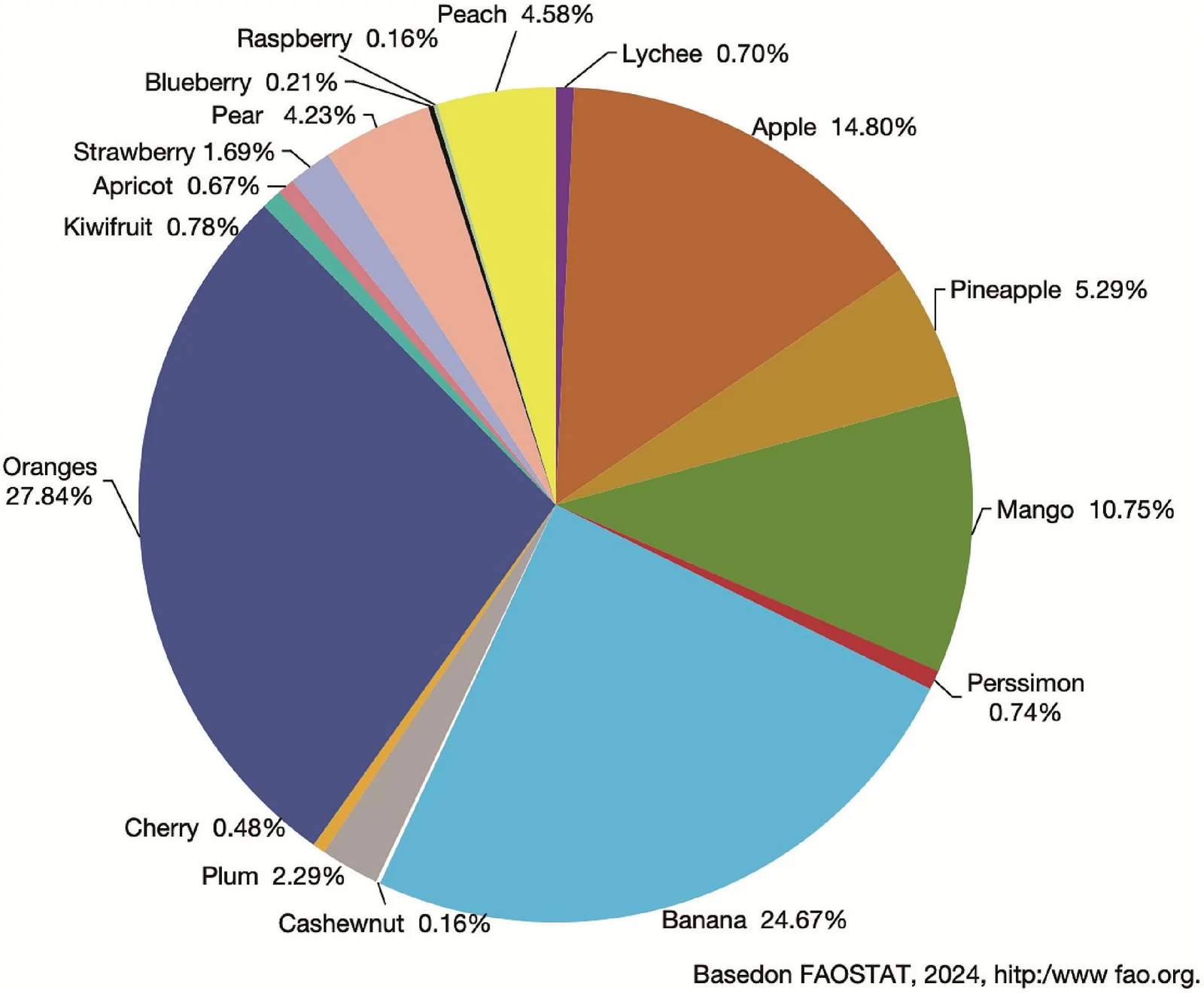
Global percentage of major non-grape fruits preferred for fruit wine production‌.

Non-grape fruit wine, known also as ‘country wines’, is an alcoholic beverage made through partial or total fermentation of fruits other than grapes. Depending on specific geographical and climatic conditions worldwide, there are ample non-grape fruits used for wine production, such as berry fruits (such as blueberry, blackberry, goji berry, strawberry, mulberry, persimmon, and pomegranate), stone fruits (such as peach, plum, apricot, mei, jujube, and cili), citrus fruits (such as orange, lemon, citron, lime and pomelo), pome fruits (such as apple, pear, loquat, and hawthorn), and tropical fruits (such as pineapple, banana, lychee, wampee, kiwifruit, papaya, pitaya, fig, and passion fruit),. In 2024, the global production of fresh fruit added up to some 951.91 million metric tons (FAOSTAT, 2024). Fig. 1 illustrated the percentage production of major non-grape fruit crops preferred as raw material for winemaking. By 2025, the global fruit wine output reached 4.8 billion liters with a market size of approaching one trillion yuan, yet 70% of publications still focus on *Vitis vinifera* L. Cider from Europe and America, perry wine from France, mei and persimmon wine from China, plum wine from Japan, and strawberry wine from Ireland are excellent examples of fruit wine and have begun to form their own cultural systems. According to different brewing types, it can be classified into distilled wine, blended wine, fermented wine, and special fruit wine (sweet fruit wine, fortified fruit wines, ice fruit wine, sparkling fruit wine, low-alcohol fruit wine, etc.). This article mainly focuses on the production of fermented non-grape fruit wine.

Terroir characteristics of different fruits determine the nutritional and flavor diversity of the raw materials, providing unlimited choices for the development of novel fruit wine products. However, its industrial fermentation faces significant challenges due to the vast diversity of fruit species and varieties, such as imbalanced sugar-acid ratios, difficulties in juice clarification, and nutrient deficiencies for yeast fermentation. Either the peel is too thick and bitter, or the flesh has too much pectin, resulting in obvious aroma loss after traditional juice preparation. While brewing presents a promising method for deep-processing non-grape fruits, improving fruit wine quality–particularly color stability, structure (body and mouthfeel), and the prominence of typical aromas–remains a bottleneck in the industry. Over the past decade, the demand for fruit wine has been changing. Consumers not only focus on its taste, quality, and nutritional value but also pursue personalized and differentiated consumption experiences, which offer new development opportunities for the fruit wine industry. Currently, the researchers still draw fermentation techniques from the grape winemaking field for fruit wine production, and the shortened cycle of international cultural and information integration leads to product homogenization of fruit wine, which undermines the personalized development of the markets.

**Fig. 2:**
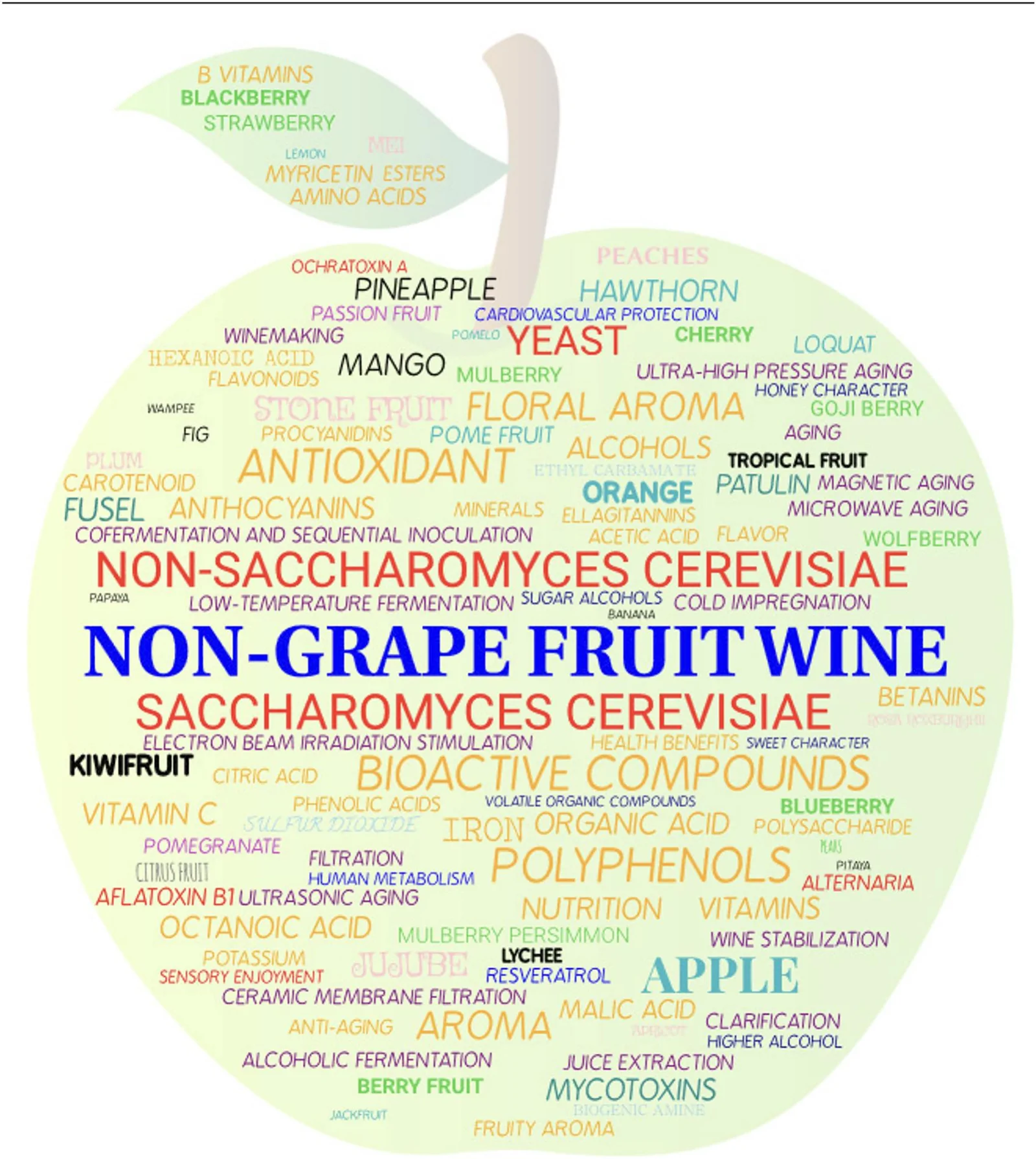
Visualization of development trends in the field of fruit wine in the past decade. The search covered publications from Dec 15, 2010 to Dec 10, 2025. The word cloud was generated using WordArt (https://wordart.com/create), based on the key search terms “non-grape fruit wine”, “non-*Saccharomyces cerevisiae”, Saccharomyces cerevisiae”*, “polyphenols”, antioxidant”, “aroma”, and “bioactive compounds”.

**Fig. 3:**
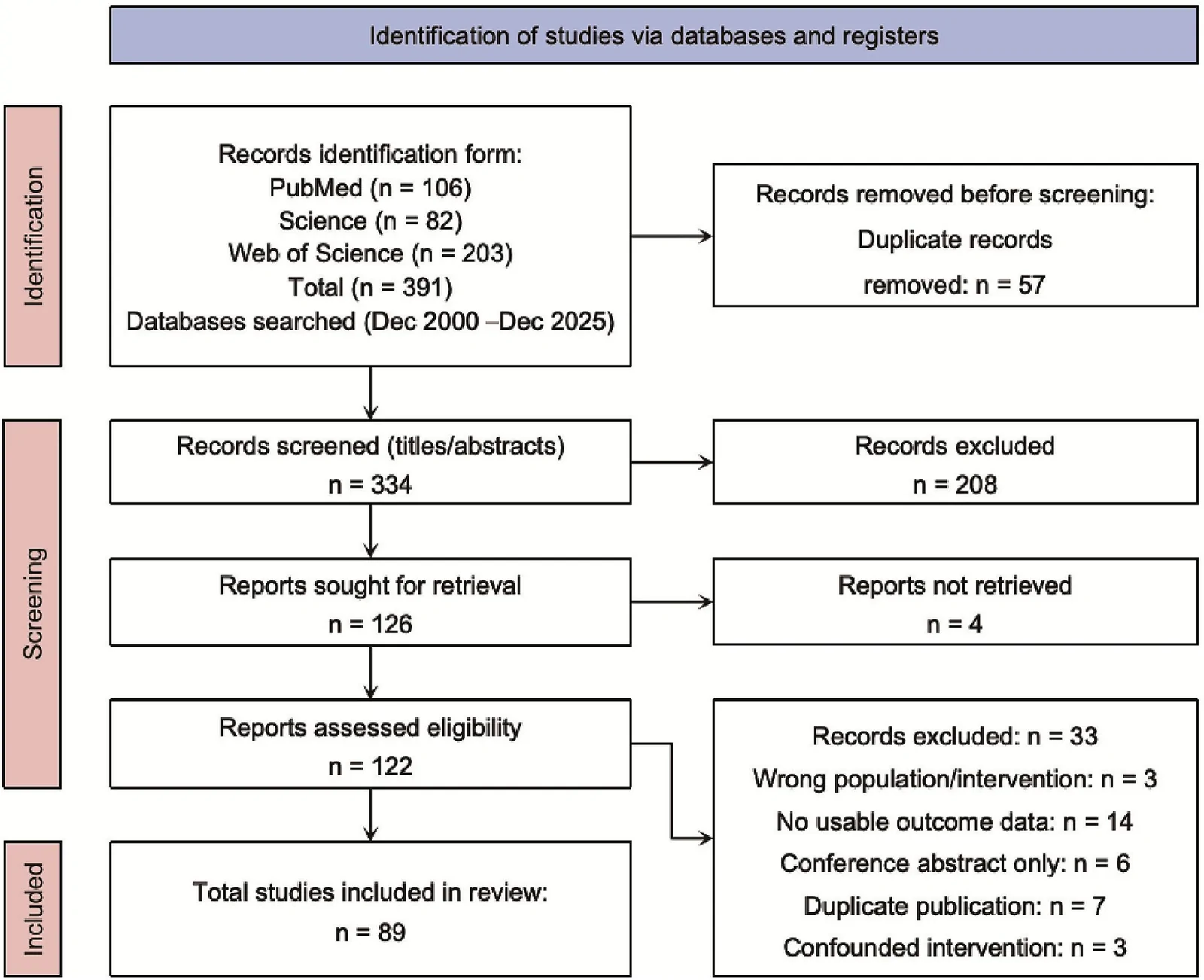
PRISMA 2020 flow diagram for study selection.

Following PRISMA guidelines, papers on the research of non-grape fruit wine from December 15, 2010, to December 10, 2025, were screened and collected through ScienceDirect, PubMed, and Web of Science. Inclusion criteria comprised studies reporting on non-grapefruit wine, phytochemicals, aroma profile, bioactive compounds, fermentation, process optimization, and safety (Fig. 2). To illustrate the current trends in non-grape fruit wine, we performed a keyword co-occurrence analysis using CiteSpace (6.1. R6) and produce ed a keyword-frequency word cloud via WordArt (Fig. 3). This article provides a detailed overview of non-grape fruit wine, encompassing its definition, varieties, healthy benefits, aroma, brewing processes and technologies, and the challenges confronting the industry during its development, aiming to give insights to support innovation in non-grapefruit wine and facilitate the upgrading of the industrial structure.

## 2 Non-grape fruits for wine production

### 2.1 Physicochemical characteristics of the non-grape fruits

**Fig. 4:**
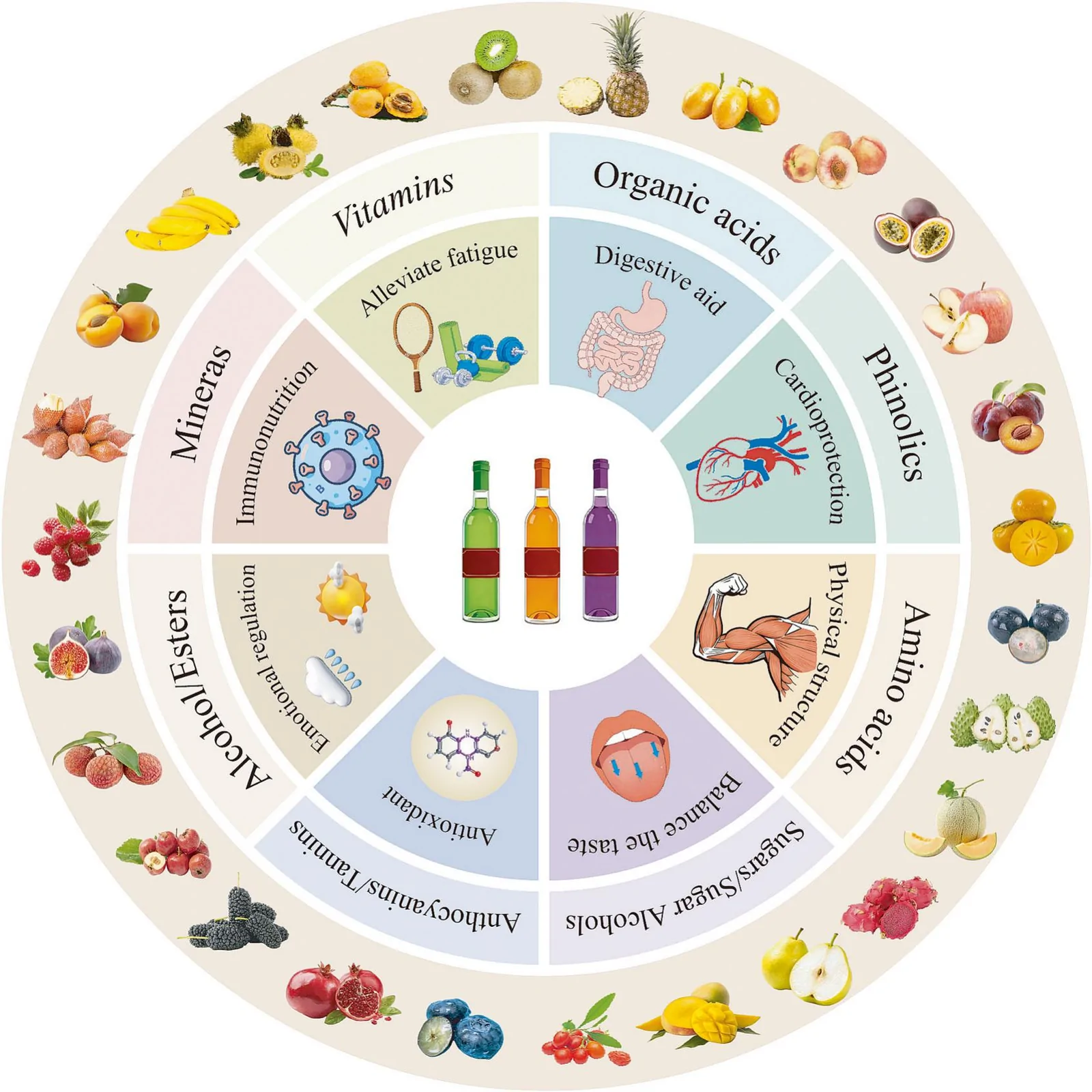
Physicochemical properties and health benefits of non-grape fruit wine.

As an important alcoholic beverage, fruit wine mainly consists of water, alcohols, and sugars and has been identified as a valuable dietary source of organic acids (citric acid, tartaric acid, acetic acid, malic acid, and oxalic acid, etc.,) trace minerals (potassium, magnesium, and iron, etc.), vitamins (mainly vitamin C and B vitamins like vitamin B_1_, B_2_, and B_12_), and carotenoids (like *β*-carotene, lutein, and xanthophylls). Notably, different fruit wines contain various phenolic compounds, which encompass anthocyanins, flavonoids (e.g., catechin, myricetin, and quercetin), proanthocyanidins, betanins, tannin, resveratrol, and phenolic acids (chlorogenic acid, caffeic acid, p-coumaric acid, ferulic acid, sinapic acid, etc.) (Maksimović & Maksimović, 2017). As shown in Fig. 4, many researchers believe that moderate drinking of fruit wines may benefit human health through their digestive aid, cardiovascular protection, antioxidant activity, and anticancer effects (Bensi et al., 2025).

### 2.2 Preparation of fruit wine

**Fig. 5:**
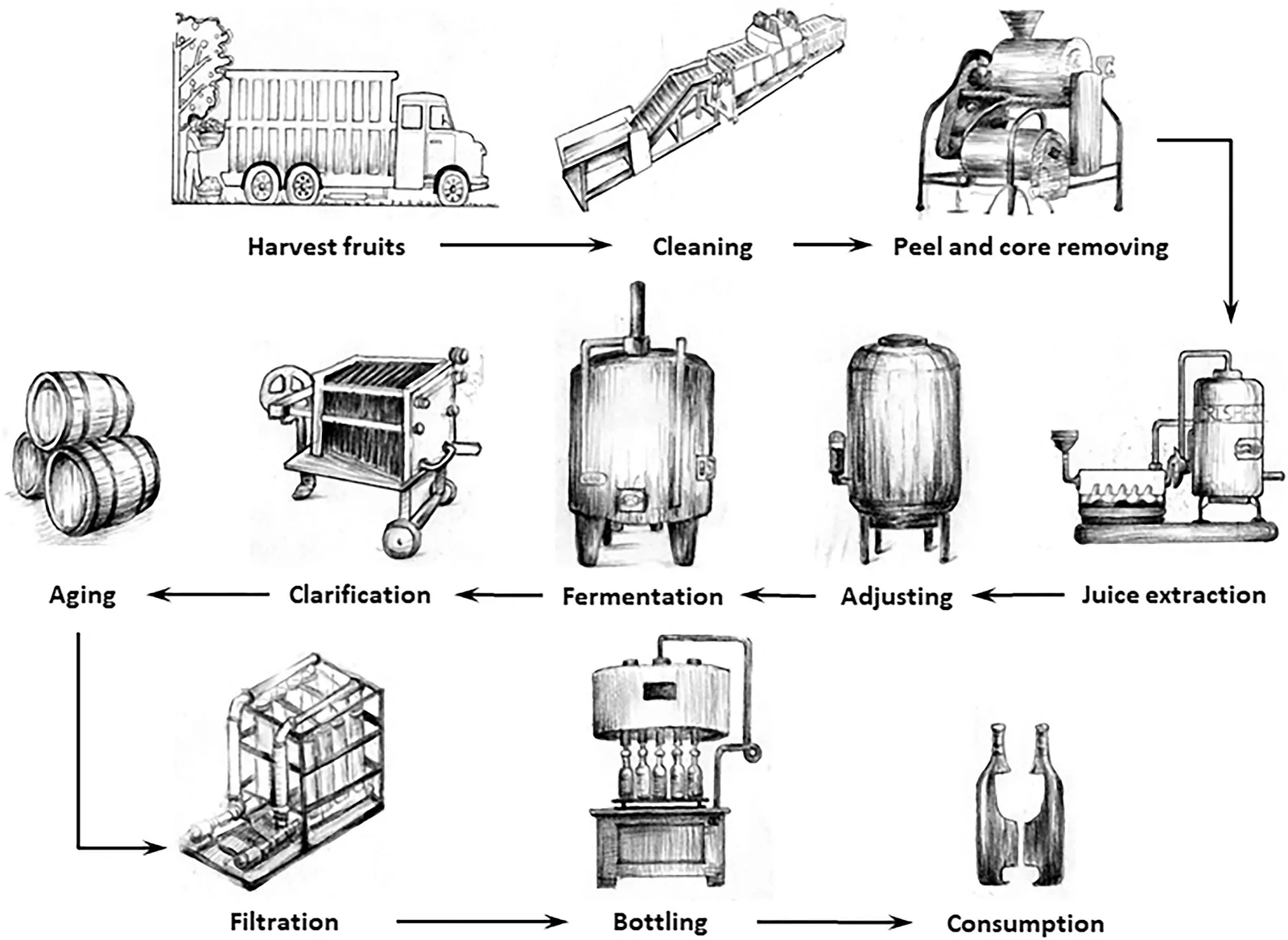
The schematic diagram of the major processing steps of fruit wine production.

The schematic diagram of the major processing steps for fruit wine production was shown in Fig. 5. Stage 1–Juice extraction. Juice prepared from different fruits varies greatly, such as berries, stone fruits, pears, and melons. Fully ripe fruits with a characteristic rich aroma are more suitable for fruit wine production, and potassium metabisulfite is evenly spread during the crushing process to prevent the growth of food spoilage bacteria. Stage 2–Alcoholic fermentation. It is a complex bioconversion process of fermentable sugars into ethanol by yeasts*.* Adjustment of sugar and acid levels prior to fermentation is a critical step to ensure successful alcoholic fermentation. The total sugar content (18–27 °Brix) and initial pH (3.0–4.0) are typically adjusted to the optimal range for yeast strains (Maheshwari et al., 2025). Stage 3–Clarification and stabilization. The fermentation is considered finish when the specific gravity is no longer decreases stops decreasing, and potassium metabisulfite is re-added to keep free sulfur at 0.4 mg/L, and then it is transferred to ceramic and stainless steel tanks for stabilization. Stage 4–Aging. After clarification, the wine will mature in earthenware, stainless steel tanks, or oak barrels. During this period, slow oxygen exposure facilitates oxidative transformations, esterification builds aromatic nuance, and phenolic polymerization enhances smoothness and taste. Stage 5–Bottling. Filtration with sterile-grade membranes is required immediately before bottling to ensure microbial stability. If necessary, inert gases (usually argon or nitrogen) are permitted to be introduced so as to form a protective blanket over the wine, shielding it from oxygen.

### 2.3 Inherent limitations in non-grape fruit winemaking

Despite their appealing sensory attributes and health-promoting phytochemicals, non-grape fruit juices present inherent deficiencies that challenge the direct application of conventional winemaking protocols, including poor processing adaptability in juice extraction, sugar-acid imbalance, and nutritional insufficiency for yeast fermentation.

#### 2.3.1 Technology and equipment challenges in juice extraction

In the pursuit of authentic, high-quality fruit wine, the initial and critical step involves the meticulous removal of skins and cores, as these components often contain bitter or astringent substances that directly impair the organoleptic quality of the final product. Unlike traditional viticulture, the processing of most tropical and non-grape fruits suffers from a lack of specialized, adaptive equipment for peeling, coring, and juice extraction, which frequently leads to suboptimal processing efficiency and compromised juice quality. For high-pectin fruits, enzymatic hydrolysis remains the predominant strategy to enhance juice yield. Enzymes such as pectinases, hemicellulases, and cellulases are widely deployed to degrade cell walls, thereby increasing yield while simultaneously reducing juice viscosity and turbidity (Stanek-Wandzel et al., 2024). However, this enzymatic approach introduces secondary biochemical challenges. For instance, pectin methylesterase (PME) can specifically catalyze the hydrolysis of methyl ester bonds in pectin, releasing methanol. Similarly, pectin acetylesterase (PAE) cleaves acetyl ester residues at the *O*-2 and *O*-3 positions of galacturonic acid, yielding acetic acid. Both metabolites can disrupt subsequent fermentation kinetics and adversely affect the sensory profile of the wine. Furthermore, aggressive pectinase treatments risk destabilizing target bioactive molecules, such as betacyanins and phenolic compounds, by inducing unwanted deglycosylation pathways (Einfalt et al., 2020; Kunnika & Pranee, 2011).

In summary, the current reliance on generic enological enzymes represents a major bottleneck for the non-grape fruit wine industry. Future research must shift from generic "juice-yielding" enzyme cocktails to customized, multi-enzyme systems tailored to the specific polysaccharide matrices of target fruits. Furthermore, the development of modular, physical pre-treatment equipment (e.g., precision destemming and flash-detartration for tropical fruits) should be prioritized alongside enzymatic engineering to mitigate the liberation of undesirable volatile precursors like methanol and acetic acid right from the start.

#### 2.3.2 Effect of non-grape fruit juice composition on fermentation process

Compared to standardized grape must, raw non-grape fruit juices present unique compositional hurdles for winemaking. A balanced sugar-to-acid ratio is a fundamental prerequisite for robust fermentation. However, many tropical fruits characterized by elevated fermentable sugars are naturally deficient in acidity, rendering the must highly susceptible to bacterial contamination throughout the fermentation lifecycle. Conversely, fruits with low sugar and high acidity present the opposite challenge: without advanced enological interventions, they yield low-alcohol products vulnerable to microbial spoilage, while residual high acidity imparts an excessively sour and astringent palate, drastically lowering consumer acceptance. (He et al., 2024).

Quantitatively, most non-grape fruits exhibit insufficient initial fermentable sugars (< 14°Brix), predominantly comprised of fructose and sucrose, which correlates to a low potential alcohol yield of only 3–8% (v/v). This necessitates exogenous supplementation with chaptalization agents like sucrose or concentrated juices to meet the standard commercial target of 10–14% (v/v) (Zhang, Hou, et al., 2025). However, poorly managed sugar additions can trigger hyperosmotic stress, severely compromising yeast viability via the repression of hexose transporter (HXT) gene expression (Türkel, 1999). Acid imbalances further complicate this matrix: low-acid musts are prone to sluggish fermentation kinetics and oxidative browning, whereas excessively high-acid environments distort sensory typicity and elevate the risk of stuck fermentations. Consequently, the initial pH of the juice is typically standardized to 3.2–3.6 using food-grade acids or weak acid salts to optimize the physiological and metabolic environment for *Saccharomyces cerevisiae*. For example, the addition of 1.0 g/L of tartaric acid can increase total acidity (TA) by approximately 1.0 g/L while depressing the pH by 0.1 units (Yang et al., 2022). Conversely, for non-grape juices dominated by high concentrations of citric or malic acids, deacidification is executed via weak acid salts, such as potassium bicarbonate or potassium carbonate. Given that the precise dosage depends heavily on the juice's intrinsic total acidity, pH, and chemical buffering capacity, rigorous, small-scale bench trials remain mandatory to prevent over-correction (AWRI (Australian Wine Research Institute), 2025; Vinmetrica, 2022).

We propose that rather than relying heavily on chemical additives (sucrose/tartaric acid), which often dilute or mask the fruit's authentic terroir, winemakers should explore ecological blending strategies. Co-fermenting high-acid/low-sugar fruits with low-acid/high-sugar tropical fruits offers a natural, clean-label approach to self-adjusting the fermentation matrix, minimizing chemical intervention while enhancing aromatic complexity.

#### 2.3.3 Effect of yeast strains on fermentation

During alcoholic fermentation, *S. cerevisiae* metabolizes fermentable sugars into ethanol and carbon dioxide, a process synchronized with the secondary synthesis of esters, higher alcohols, and volatile metabolites that fundamentally dictate the physicochemical profiles, sensory traits, and consumer appeal of the final wine (summarized in Table 1). At present, conventional commercial *S. cerevisiae* strains dominate industrial-scale fruit winemaking. However, their metabolic uniformity frequently generates homogenized aroma profiles dominated by recurrent concentrations of standard compounds such as 3-methylbutanol, ethyl acetate, and isoamyl acetate. This aromatic homogeneity ultimately thins the mouthfeel, constrains complexity, and dilutes product typicity, causing the resulting wines to fail to express the distinctive, varietal features of the ancestral fruit. (Lu et al., 2021). Therefore, the isolation of specialized, non-grape yeast strains and the precise tailored refinement of fermentation protocols remain urgent priorities for the industry. Simultaneously, rigorous yeast nutrition management is indispensable for ensuring seamless fermentation kinetics. Specific enological aids are regularly required to supplement bioavailable organic and inorganic micronutrients—including vitamins, minerals, and amino acids—to accelerate yeast acclimatization and maintain high cell viability, particularly in demanding fruit musts. In industrial practice, managing Yeast Assimilable Nitrogen (YAN) is paramount to stimulate robust biomass accumulation and avert the synthesis of off-flavor volatile compounds, such as hydrogen sulfide (H_2_S), which stems from disrupted sulfur assimilation pathways (Bisson & Butzke, 2000). It has been well-documented that YAN levels falling below 140 mg/L drastically escalate synthesis; crucially, the ideal strategies for exogenous nitrogen supplementation must be dynamically tailored to the specific fruit type and the unique metabolic demands of the selected yeast strain (Bekker et al., 2023).

**Table 1:** List of brewing programs and properties of main non-grape fruit wines.

Fruit types	Strains	Temperature and time	Alcohol *%, v/v*	Antioxidative characteristic	References
Apricot (*Prunus armeniaca* L.)	*S. cerevisiae* CEC01	20°C for 8 d	11.4	TPC 289.9 mg GAE/L/L, TFC 19.05 mg RE/L/L, DPPH-RSC 68 mg TE/L, ABTS-RSC 90 mg TE/L, FRAP-RSC 35 mg TE/L	Pu et al., 2023
Blueberry (*Vaccinium corymbosum*)	*S. cerevisiae* NCUF309.2 *Candida glabrata* E4	26 °C for 7 d, and continued at 20°C for 15 d	8–10	TPC 0.87–1.09 g GAE/L, TFC 0.42–0.58 g RE/L	Che et al., 2024
Wild cherry (*Prunus cerasus* L.)	*H. uvarum* YT-35, *S. cerevisiae* SC-125	28 °C for 5d		TPC 5936.60 mg GAE/L, DPPH-RCS 32.93 mmol TE/L, ABTS-RCS 136.52 mmol TE/L, ·OH-RSC 48.29 mmol TE/L	Peng, Zhang, et al., 2025
Cashew (*Anacardium occidentale* L.)	*Hanseniaspora opuntiae* 125–LPMA 2023, *Issatchenkia terricola* 129–CCMA 2040	20°C for 8–11 d	6.37–11	T-OAC 9.81 mg/L, TPC 30.06–42.60 mg/L	Amorim et al., 2026
Cili (*Rosa roxburghii* Tratt)	*S. cerevisiae* BV818 and D254	18°C	9.9–12.2		Lei et al., 2024
Kiwi (*Actinidia*)	Yeast	20°C for 8 d		TFC 230–520 μg GAE/g, DPPH-RSC 1.41–5.28 μmol TE/L, FRAP-RSC 13.32 μmol TE/L	Wei et al., 2025
Kiwi (*Actinidia*)	*Zygosaccharomyces rouxii* 1130, *S. cerevisiae*	18°C for 14 d	3.83–6.87	TPC 975.49 mg GAE/L, TFC 96.00 mg GAE/L, ·OH-RSC 85.20%, DPPH-RSC 54.55%, ·O_2_^—^RSC 73%, FRAP-RSC 2.27 mmol TE/L	Li et al., 2023
Jujube (*Ziziphus jujuba* Mill.)	*S. cerevisiae* BV818	20°C for 10 d	12.4–12.9	TPC 1069.73 mg GAE/L, TFC 629.82 mg RE/L, DPPH-RSC 85-91%, ABTS-RSC 35-88%	Hao et al., 2025
Litchi (*Litchi chinensis* Sonn.)	*S. cerevisiae* EC-1118	25°C	12		Zhao et al., 2021
Uvaia (*Eugenia pyriformis* Cambess)	*S. bayanus*	20°C	3.71–4.05	TPC 248.83-337.03 mg GAE /L, FRAP-RSC 733.8 μmol TE/L, ABTS-RSC 3009 μmol TE/L	Ferreira et al., 2024
Mei *(Prunus mume)*	Yeast	28°C for 56 h	6.75	TPC 10 mg GAE/L, TFC 1500 mg GAE/L, T-OAC 4 mg CGE/L, DPPH-RSC 81%, ABTS-RSC ∼99%	Sui et al., 2024
Greengage *(Prunus mume)*	*S. cerevisiae,* Lalvin71B	22 ± 1 ° for 10	8.53–12.72	TPC 33.89 mg GAE/L, TFC 343.54 mg RE/mL, T-OAC 56.40 U/mL	Han et al., 2020
Loquat [*Eriobotrya japonica* (Thunb.) Lindl.]	*S. cerevisiae* PF-6	22–26°C for 12 days	12.5		Liu et al., 2024
Sea red fruit (*Malus micromalus Makino*)	*S. cerevisiae* VL2		10	DPPH-RSC 97.07%, ·OH-RSC 80.36% ·O_2_^-^-RSC 94.12%	Yang et al., 2021
Raspberry (*Rubus occidentalis*)	*S. cerevisiae* Authentique, *Torulaspora delbrueckii* Biodiva	24–26°C for 6–9 d	8.6–11.1	TPC 6.08 mg GAE/g, TAC 2.43 mg CGE/g, DPPH-RSC IC_50_ 221 μg/mL, ABTS-RSC IC_50_ 295 μg/mL	Jeong et al., 2010
Mulberry (*Morus alba* L.)	*S. cerevisiae*	22–24 °C for 7–8 d		TPC 14.00 g Vc/L, TFC 1.25 g RE/L, TAC 400 mg CGE/L, ABTS-RSC 91.92%, DPPH-RSC 92.50%	Bian et al., 2025; Lian et al., 2024
Strawberry *Fragaria* × *ananassa*	*S. cerevisiae* EC-118	22–24°C for 10 d	12.17–12.50%	TPC 500-790 mg GAE/L, TFC 130-340 mg RE/L, TAC 2.79–14.41 mg CGE/L, DPPH-RSC 100-500 mg AEAC, ABTS-RSC 200-620 mg AEAC	Lv et al., 2024
Persimmon (*Diospyros lotus* L.)	*S. cerevisiae* Fermivin	25°C for 7	7.93	TPC 668.10 mg GAE/g, TFC 56.99 mg RE/g, DPPH-RSC IC_50_ 41.79 μg TE/mL, ABTS-RSC IC_50_ 41.79 μg TE/mL, FRAP-RSC IC_50_ 2.54 g FeSO_4_/g	Jang et al., 2025
Pomegranate (*Punica granatum* L.)	*S. cerevisiae* Clos, *S. cerevisiae ex-bayanus* EC1118	16–24°C for 10 d		TPC 1588-1924 mg GAE/L, T-OAC 87–300 mg/L	Cardinale et al., 2021
Banana (*Musa acuminata* L.)	*S. cerevisiae* K1-V1116	25°C for 30 d	12–16	TPC 3.50 mg GAE/g, TFC 4.74 mg/g, DPPH-RCS 90–100%	Li et al., 2023
Mango (*Mangifera indica* L.)	*S. bayanus* Lalvin EC 1118	20 ± 2°C for 18 d	13		Kamran et al., 2025
*Passion* *(Passiflora cincinnata Mast.)*	*S. bayanus*	24±2 for 4–6 d	6.99–8.12	TPC > 0.7 g GAE/L, DPPH-RCS 2.78 mmol TE/L, FRAP-RCS 7.61 mmol TE/L, ORAC-RCS 15.412 mmol TE/L	Santos et al., 2021
Karonda (*Carissa carandas* L.)	*S. cerevisiae* MTCC 11815	28°C for 5 d	4.55–5.56	TPC 150–179 mg GAE/L, DPPH-RCS 81.05–84.85 %	Arora et al., 2023
Pineapple (*Ananas comosus*)	*S. cerevisiae Brettanomyces lambicus, Brettanomyces bruxellensis, Lachancea thermotolerans*	20°C for 13 d	*9.78*–*12.4*	*TPC 45.86–55.32 mg* GAE/L	Anjos et al., 2024
Plum (*Prunus salicina*)	*S. cerevisiae* Lalvin EC1118	20°C for 7 d	13.1		Liu et al., 2021
Palm (*Elaeis guineensis* and *Raphia regalis*)	*S. cerevisiae* SC-22	28-30°C for 12 d	12–13		Khadka et al., 2024
Apple (*Malus domestica*)	*S. cerevisiae* 32168	20 ± 1°C for 7–10 d	6.29–7.61	TPC 0.41 mg/mL to 0.71 mg/mL, TFC 0.33–0.89 mg/mL, T-OAC 42.69–91.85 U/mL, ·OH-RSC 6.93 %–17.90 %	Zeng et al., 2024
Goji berry (*Lycium barbarum*)	*S. cerevisiae* RW	20 for 8 d	8.80	TFC 1.22 g GAE/L, DPPH-RCS 1.27–1.87 g TE/L, ABTS-RCS 1.61–2.13 g TE/L	Peng et al., 2025
(*Ficus carica*)	*S. cerevisiae* RV171	28°C for 10 d	12	TPC 282.58 mg GAE/L, TFC 110.61 mg GAE/L, DPPH-RSC 75.76%, ·OH -RSC 85.72%	Ma et al., 2022
Navel oranges (*Citrus sinensi*s)	*S. cerevisiae* native yeasts	10 and 20°C		TPC 6.9–11.9 GAE/L, TFC 31.08–50.20 mg GAE/L, DPPH-RSC 39.20 AEAC, ABTS-RSC 2.54–28.23 AEAC, FRAP-RSC 0.07–0.14 FeSO_4_ μg/mL)	Schvab et al., 2015
Pitaya (*Hylocereus undulatus*)	*S. cerevisiae* Z03M *Torulaspora delbrueckii*	25°C for 14 d, 28°C for 7 d	7.75-8.0	TPC 302.83 mg GAE/L, BC 112.24 mg BE/L, TAC 9.99 mmol TE/L, FRAP-RSC 3.58 mmol/L, DPPH-RSC 80.2%	Jiang et al., 2020; Wang, Liu et al., 2024
Wampee (*Clausena lansium*)	*S. cerevisiae*	21–23°C for 9 days	11		Wang et al., 2024
Kei apple (*Dovyalis caffra*)	*S. pombe* V2 and *S. cerevisiae* NT116	22°C for 10 d	3.1–3.3		Minnaar et al., 2021
Papaya (*Carica papaya*)	*Williopsis saturnus var. mrakii* NCYC 2251	20 for 21 d	9		Lee et al., 2011
Pear (*Pyrus communis* L.)	*S. cerevisiae*	26°C for 10 d	6.4–6.6	TPC 300 GAE/L, TFC470 mg RE/L, AEAC 6.7 mmol TE/L, TYR 52%, FRAP-RSC 3.1 mmol TE/L	Yang M, et al., 2025
Wax apple (*Syzygium aqueum*)	*S. bayanus*	25°C for 10 d	6.5	TPC 214.44 μg/mL, TFC 291.87 μg/mL, DPPH-RSC 84%, ABTS-RSC 62%, ·OH-RSC 69%	Karthikeyan et al., 2018
Peach (*Prunus persica* L.)	*S. cerevisae* Aroma White	17°C for 14 d	8.12	TPC 403 mg GAE/L, TFC 333 mg CAE/L, DPPH-RSC 1.55 mmol TE/L, FRAP-RSC 3.01 mmol TE/L	Davidović et al., 2013

Note: Total phenolic content (TPC), Total flavonoid content (TFC), Total anthocyanin content (TAC), Total antioxidant capacity (T-OAC), Ascorbic acid equivalents antioxidant activity (AEAC), Betacyanin content (BC), Betanin equivalent (BE), cyanidin-3-*O*-glucoside chloride equivalent (CGE), Gallic acid equivalents (GAE), Rutin equivalents (RE), Trolox equivalents (TE), Tyrosinase inhibition activity (TYR), DPPH radical scavenging capacity (DPPH-RSC), ABTS radical scavenging capacity (ABTS-RSC), Hydroxyl radical scavenging capacity (·OH-RSC), Superoxide radical scavenging capacity (·O_2_-RSC), FRAP radical scavenging capacity (FRAP-RSC).

We argue that the commercial failure of many fruit wines to capture premium markets is directly linked to this "aromatic homogenization" caused by standard industrial wine yeasts. To unlock the true potential of non-grape fruit wines, future research must move away from monocultures of S. cerevisiae. We highlight the immense potential of utilizing indigenous non-Saccharomyces yeasts (e.g., *Torulaspora delbrueckii* or *Lachancea thermotolerans*) in sequential or co-inoculation regimes. These non-conventional yeasts can naturally modulate high acidities, liberate fruit-bound glycosidic aromas, and establish a distinct sensory identity that distinguishes fruit wines from cheap imitations of grape wines.

#### 2.3.4 Effect of temperature on fermentation properties

The kinetic and metabolic expressions of *S. cerevisiae* are highly sensitive to ambient fermentation temperatures. Under low-temperature regimes, microbial metabolism transitions into a prolonged, gradual, and thorough kinetic profile, facilitating a more controlled conversion of sugars and profoundly modifying the ratio and composition of organic acids, alcohols, and volatile flavor fractions. Specifically, low-temperature fermentation (5–16°C) down-regulates glycolytic velocity, reduces the accumulation of volatile acidity (acetic acid), and suppresses the biosynthesis of heavy fusel alcohols compared to warm fermentations (25–32°C). This thermal deceleration concurrently creates thermodynamic and metabolic environments that favor the synthesis and retention of delicate, ester-derived volatile profiles, notably ethyl isovalerate and ethyl acetate (Massera et al., 2021). Conversely, elevated temperatures accelerate metabolic flux and boost the enzymatic catalytic efficiency of specialized strains, such as *S. cerevisiae* AP05 during apple winemaking, significantly driving the accumulation of key ester and alcohol groups including ethyl acetate, isoamyl acetate, ethyl caprylate, and 2-phenylethanol (Peng, Huang, et al., 2025). Temperature also exerts a profound impact on the structural preservation of the raw material’s inherent attributes. For example, during blueberry winemaking, a cooler fermentation temperature (17°C) markedly improves the retention of monomeric pigments, bioactive fractions (such as vitamin C, tannins, flavonols, and anthocyanins), and overall antioxidant capacities compared to a 21°C (Varo et al., 2024). Ultimately, temperature dictates not merely the kinetic velocity, but the survival of volatile heritage; managing fermentation at 10°C has been shown to boost the accumulation of ethyl octanoate and fatty acid ethyl esters, correlating with a 14% increase in descriptive sensory scores (Shi et al., 2022).

In conclusion, fruit wines heavily rely on low temperatures (10–15°C) to lock in volatile, heat-sensitive esters, berry-based wines (e.g., blueberry or blackberry) require a dynamic, multi-stage temperature profiling strategy. We propose implementing an innovative "warm-start, cold-finish" thermal regime: an initial warm phase (20–22°C) for brief skin-contact to maximize the extraction of polyphenols and pigments, followed by a rapid chill to low temperatures (12–14°C) to preserve the ester profile and prevent oxidative color loss.

#### 2.3.5 Trade-off between stability and quality

The stabilization of non-grape fruit wines is still an unavoidable challenge, particularly for juice matrices characterized by water-soluble phenolic compounds (e.g., anthocyanins, flavanols). These constituents readily facilitate the formation of polyphenol–protein and polyphenol–polysaccharide complexes, which are initially soluble but progressively aggregate during storage, ultimately resulting in haze formation and sedimentation upon bottling (Liu, Liu, Qin, et al., 2025). For high-phenolic fruit wines (e.g., blackberry, blueberry, and elderberry), cold stabilization and/or fine filtration strategies can mitigate haze-forming precursors during aging (Wu et al., 2021). Achieving an optimal balance between preserving the characteristic aroma of raw material and ensuring adequate stability remains technically challenging.

## 3 Quality improvement of non-grape fruit wines

### 3.1 Improvement of the juice quality by pretreatment

Glycosidase can transfer disaccharide glucosides into monosaccharide glucosides, which are important natural aroma precursors in non-grape fruit, like terpenoids, aromatic esters, higher aliphatic alcohols, etc. (Lv et al., 2024). The fruit juice (apple, apple-raspberry, and apple-mango) treated with commercial glycosidase showed a significant release of new aroma compounds. For example, 2-methyl butyric acid, chavicol, and eugenol were released in the apple-based juices with the enzymatic treatments, indicating that they are potential aroma precursors bound in monosaccharide glucosides in untreated apple juice (Schmidt et al., 2025). However, glycosidase can diversify the flavor of fruit juice but is not yet widely employed in standard fruit wine fermentation, which is related to the unpredictable effect on the overall aroma metabolism during fermentation and the higher production costs.

Maceration treatment (MT), mainly involving carbonic or nitrogen maceration, cold soaking, extended maceration, etc., demonstrated distinct advantages in extracting key components (thiols, terpenes, anthocyanins, etc.) from fruit skins and reducing the concentration of C6 alcohols and some higher alcohols, contingent on strain selection and process optimization. (Guo et al., 2025). In berry fruit wine production, cold maceration treatment (below 15°C) prior to fermentation is successfully employed for extraction of phenolic compounds related to the color and flavor, providing a promising solution for enhancing the fruit wine quality (Sperotto et al., 2024). However, those phenolic from MT might interfere with the growth and metabolism of yeast through interaction with cell membranes, thus influencing the yeast growth and fermentation process, so practical MT techniques for large-scale fruit wine production are still missing (Du et al., 2024).

### 3.2 Aroma enhancement driven by microorganism strains

The selection of strains and inoculation protocols plays an important role in the production of fruit wines. Indigenous non-*Saccharomyces* yeasts possess distinctive enzymatic profiles. For instance, *Metschnikowia pulcherrima* expresses high *β*-glucosidase and *β*-lyase activity, hydrolyzing glycosylated aroma precursors to release free terpenes and thiols that enhance fruity and floral notes (Bertazzoli et al., 2025; Guo et al., 2025).

Non-*S.* yeasts, like *L. thermotolerans, Hanseniaspora uvarum*, and *T. delbrueckii*, have high β-glucosidase activity and exhibit notable advantages in improving the aromatic complexity of fruit wines by releasing volatile compounds from glycosylated precursors and increasing ester production (Tang et al., 2025; Zhang, Hou, et al., 2025). In goji berry wine fermented by *L. thermotolerans,* fruity, honey, and goji berry aromas were enhanced, while off-flavors induced by 4-ethylguaiacol decreased by 50%, and free flavonoids of quercetin and morin, phenolic acids, and phenolamines increased significantly (Zhu et al., 2025). In pineapple wine, *L. thermotolerans* produced 0.95 g/L of lactic acid, generated pineapple aroma-related ethyl butyrate (260–270 μg/L), improved phenolic bioaccessibility (catechin by 237%), and enhanced fruity esters compared to conventional S. cerevisiae fermentation (Anjos et al., 2024). However, these yeasts usually showed limited fermentative capacity and weak alcohol tolerance compared to S. cerevisiae, usually producing 4–6% of ethanol, with some strains reaching up to 9–10% (Zhang, Lv, et al., 2025). In dragon fruit wine, *L. thermotolerans* with lactate dehydrogenase activity conversed pyruvic acid to lactic acid, thereby lowering the pH and promoting the formation of the flavylium cation of betacyanin, which helps maintain the red intensity. However, *T. delbrueckii* produces minimal acetic acid and acetaldehyde, resulting in preservation of betacyanin and phenolic content, which contributed significantly to the antioxidant capacity of dragon fruit wine (Jiang, Lu and Liu, 2020a, Jiang, Lu and Liu, 2020b).

Co-fermentation of *S. cerevisiae*‌ and non-*S.* cerevisiae was widely adopted to improve aroma formation and accumulation in numerous fruit wines (Schober et al., 2023). ‌Sequential inoculation of *L. thermotolerans,* and followed by *S. cerevisiae* after 24–72 hours, has been shown to modulate aromatic profiles in goji berry wine (Zhu et al., 2025). *Metschnikowia pulcherrima* and *S. cerevisiae* were able to increase total ester content and enhance the abundance of key aroma compounds such as isoamyl acetate (fruity, banana), terpenols (floral, citrus and woody), and phenylethyl alcohol (floral, rose), when used in sequential ‌inoculation during strawberry wine production. Sequential fermentation with *M. pulcherrima* and *L. thermotolerans* elevates ethyl hexanoate levels and enhances fruity and floral notes in strawberry wine. This effect stems from elevated β-glucosidase activity, which hydrolyzes glycosylated aroma precursors (Xu et al., 2025). Comparable mechanisms operate in kiwifruit wine fermented with non-*Saccharomyces* yeasts and in apple wine produced with *M. pulcherrima* (Kregiel et al., 2022; Sun et al., 2024). Screening of indigenous yeasts and refinement of fermentation protocols remain priorities in oenological research. Sequential strategies show considerable promise for enhancing aromatic complexity and mouthfeel (Wang et al., 2023). Their efficacy in non-grape fruit matrices, however, varies with strain identity and remains partially unpredictable, warranting further systematic investigation.

### 3.3 Influence of volatile metabolism under low-temperature fermentation

The aroma components of fruit wine mainly involved fruit-derived aroma, fermentation-derived aroma, and maturation-derived aroma. Among various environmental factors, temperature exerts a critical effect on both the speed and duration of the fermentation process (Massera et al., 2021). Unlike high-temperature fermentation (25–32°C), low-temperature fermentation (10–20°C) can weaken glycolysis, decrease acetic acid production, and suppress fusel alcohol formation, while facilitating the synthesis of aroma compounds such as ethyl acetate and ethyl butyrate (Du et al., 2022). Fermentation with non-*Saccharomyces* yeasts at 18°C has been shown to enhance polyphenolic retention, increase ester production, and improve antioxidant capacity and flavor profile in strawberry wine (Wang et al., 2025). Blueberry wine fermented at 17°C retained higher vitamin C content and showed better preservation of bioactive compounds (Varo et al., 2024). However, Low-temperature fermentation requires YAN supplementation for normal yeast growth and metabolism (Bisson & Butzke, 2000).

## 4 Colloidal and color stability during post-fermentation

### 4.1 Haze formation mechanism in non-grape fruit wine

**Fig. 6:**
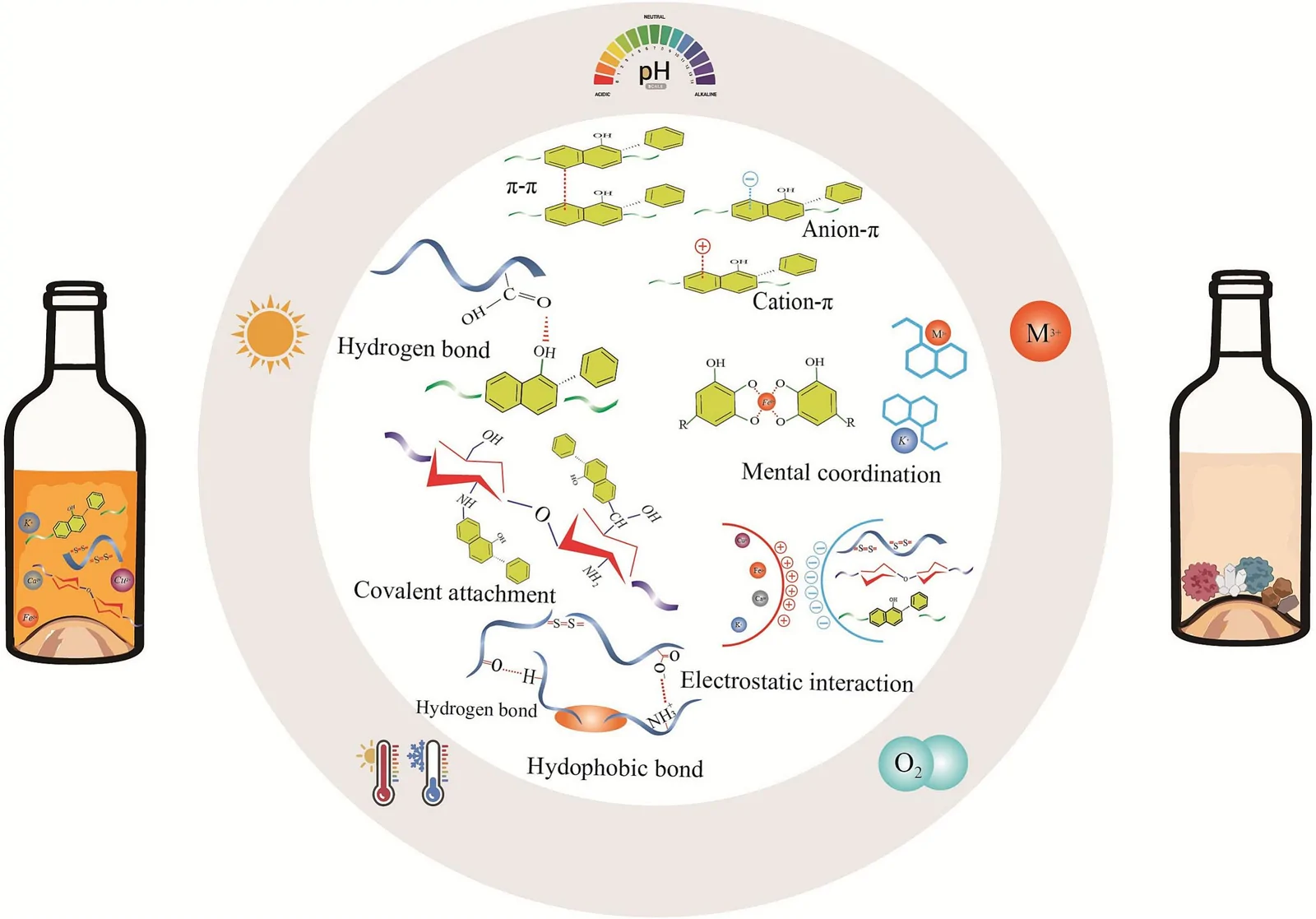
The major mechanism of haze formation in fruit wine.

As dominant sensory defects, haze and secondary precipitation are quality issues in the fruit wine industry. Fig. 6 presents the mechanism on the formation of primary haze in most fruit wines. Generally, the interaction of polyphenols and proteins driven by weak intermolecular interactions such as hydrogen bonding and/or hydrophobic interactions is key cause for non-grape fruit wines, and only a few of the polyphenols bind with protein polyphenol and subsequently generate large cross-linking. Of course, the denaturation of heat-labile proteins could also cause flocculation by protein cross-linking. In addition, polyphenolic compounds such as anthocyanins and tannins can also aggregate by self-linking or complexation with other large molecules. During the maturation of blackberry wine, protein aggregation is not the main factors of the formation of haze; it is the polymerization of polyphenols (200–500 m/z) and proteins (20–100 kDa). Moreover, metal ions such as K and Fe ions are proved to accelerate the aggregation of proteins and polyphenols by hydrogen bonding (Wu et al., 2024). During the period of storage of fruit wine, those initially soluble complexes are gathering as large insoluble molecules, namely visible haze, and this process has been accelerated in multiple fruit matrices with pectin, arabinogalactan, and polygalacturonic acid, while soluble carbohydrates and free amino acids showed no effect. Additionally, the most obvious haze phenomenon occurs at a pH of 4, and there is a non-linear relation between ethanol concentration and haze.

Turbidity and precipitation in fruit wine exhibit distinct stage-specific characteristics. Initially, proteins experience reduced solubility and precipitate due to temperature fluctuations and isoelectric point effects (Siebert et al., 1996). Subsequently, endogenous polyphenolic compounds (e.g., anthocyanins and condensed tannins) engage in hydrophobic interactions, hydrogen bonding, and metal ion complexation (with Fe^3+^, K^+^, etc.) to form nanoscale soluble complexes (Teng et al., 2021). These complexes then undergo cross-linking and aggregation mediated by polysaccharide macromolecules (such as gum and arabinogalactan) (Jaeckels et al., 2016), while polyphenols simultaneously undergo oxidative polymerization, yielding submicron-sized unstable colloidal particles (Teng et al., 2021). Ultimately, the colloidal system destabilizes, resulting in visible precipitates. Additionally, the solubility of tartaric acid in alcohol is lower than in fruits. When fruit wine contains high concentrations of metal cations (e.g., calcium and potassium), they can combine with tartaric acid to form insoluble tartrate crystals, which precipitate out of solution (Cui et al., 2024).

Turbidity and precipitation in fruit wine can be addressed through interventions at three distinct stages. At the molecular level, pH adjustment prevents proteins from reaching their isoelectric point, thereby minimizing aggregation (Van Sluyter et al., 2015). Proteases reduce protein molecular weight and aggregation propensity (Marangon et al., 2012; Zhao et al., 2024). Chelating agents such as EDTA sequester metal ions (Fe^3+^, K^+^), attenuating their catalytic role in polyphenol oxidation and protein cross-linking (Waterhouse & Laurie, 2006). During colloidal aggregation, membrane filtration achieves physical separation based on particle size (Sui et al., 2021, Sui et al., 2022). Hydrocolloids, like gum arabic, generate steric hindrance and could inhibit particle growth (Lankhorst et al., 2017). Antioxidants such as ascorbic acid scavenge free radicals and prevent oxidative polymerization of polyphenols (Danilewicz & Wallbridge, 2010). At the phase separation stage, filling protocols and storage parameters (temperature, oxygen exposure) are rigorously controlled to delay precipitation onset (Cui et al., 2024). In conclusion, effective stabilization requires integrated intervention across all three scales, blocking the progression from molecular interactions to macroscopic phase separation.

### 4.2 Browning inhibition in non-grape fruit wine

Oxidative browning is a long-standing problem in fruit wine production and seriously affects the quality of fruit wine. For most light-colored wines, mild oxidation exposure is beneficial to promote anthocyanin-tannin polymerization, which enhances color stability. However, excessive oxidation always causes degradation of anthocyanins and subsequently induces browning (Li, Zhang, et al., 2025). Polyphenol oxidase (PPO), as an important oxidoreductase, could catalyze *O*-diphenols to highly reactive *O*-quinones, which polymerize and produce melanin and are responsible for enzymatic browning in strawberry wine. PPO activity declines as ethanol accumulates during fermentation, non-enzymatic browning via phenolic oxidation and Maillard reactions is predominant (Zavarise et al., 2025; Zhao et al., 2023). It was proved that addition of antioxidants, as well as control of temperature and dissolved oxygen levels during fermentation can effectively control browning in fruit wine (Wang, Liu, et al., 2024). Glutathione (*γ*-glutamyl-cysteinyl-glycine; GSH) serves as a crucial antioxidant that can mitigate oxidative browning by blocking the formation of free radicals (e.g., orthoquinone), scavenging reactive oxygen species (ROS), and chelating metal ions due to its low redox potential (superior to that of ascorbic acid). Moreover, it can protect aromatic compounds and reduce the reliance on SO₂ (Bustamante et al., 2025). Zhang et al. (2024) found that 400–800 mg/L of GSH significantly inhibits oxidative browning in cili wine production, and its antioxidant capacity surpasses that of sulfur dioxide at equivalent doses. *S. cerevisiae* strains with high glutathione synthesis capacity significantly inhibit browning while demonstrating good fermentation performance during the pear wine fermentation (Yang, Wang, et al., 2024. Bustamante et al. (2023) reported that *Metschnikowia pulcherrima* prevented oxygen-induced enzymatic browning by rapidly consuming dissolved oxygen in fruit wine, offering a promising alternative to SO₂. In addition, stainless steel tanks with lower oxygen permeability compared whit ceramic jars weakens oxidative and non-enzymatic browning. During the aging period, stainless steel tanks retain higher levels of monomeric anthocyanins and delay the formation of polymeric pigments from anthocyanins compared to those aged in oak barrels, thereby maintaining excellent color stability (Guerrini et al., 2022; Pichler et al., 2024). Nonetheless, there is always a lack of unified measures for preventing browning due to differences in raw materials used for fruit wine production.

### 4.3 Traditional and potential innovative techniques for fruit wine aging

In conclusion, the fruit wine in a bottle or oak barrel gradually becomes more stable, mellow, complex, and easier to drink during the natural aging process. Natural aging can enhance the wine's quality, but it requires significant time and extends the production cycle. Artificial aging techniques including traditional oak aging, microwave aging, ultra-high pressure aging, ultrasonic aging, electron beam irradiation stimulation, and magnetic aging) can accelerate the maturation process, as well as improve flavor and functional characteristics and production efficiency. However, each technique has specific application conditions and risks, so selection and control should be based on the fruit type and quality of the specific fruit wine (Liu, Liu, Li, et al., 2025). Based on the literature available, those new technologies displayed good potential in wine aging, but they have not been included among the enological practices recommended by the International Organization of Vine and Wine (OIV), and most of their enological applications still need to be investigated at pilot-plant scale.

## 5 Potential safety and risk in fruit wine production

### 5.1 Mycotoxin contamination

Mycotoxins are toxic secondary metabolite produced by various fungal genera and can be generated throughout the entire fruit lifecycle, including growth, harvesting, storage, and processing. Physically damaged or pathogen-infected fruits are particularly susceptible to contamination by mycotoxin-producing fungi such as *Penicillium*, *Byssochlamys*, and *Aspergillus*. Tropical fruits (excluding grapes) grown in high-temperature, high-humidity environments face significantly higher contamination risks (Kamle et al., 2022; Nan et al., 2022; Shen et al., 2025). Table 2 summarized the potential mycotoxins contaminated in raw materials for non-grape fruit and its products. Fungicides effectively control preharvest mold but introduce new risks to food safety, ecosystems, and human health. Compounding this challenge, mycotoxins resist most physicochemical processing, leaving physical removal as the primary practical intervention. This includes trimming decayed tissue and thorough washing. In products like juices and concentrates, detectable mycotoxin levels often point to lapses during manufacturing rather than simply reflecting raw material quality. Adopting good manufacturing practices, such as stringent fruit selection and rigorous cleaning, can effectively reduce residual mycotoxin levels.

**Table 2:** Potential mycotoxins contaminated in raw materials of non-grape fruit wines.

Mycotoxins	Primary sources	Products	References
Citrinin	*Penicillium expansum* *P. citrinum*	Apple, pear wines, pineapple juices	(Kamle et al., 2022; Shen et al., 2025)
Patulin (PAT)	*Byssochlamys fulva* *B. nivea* *P. expansum*	Apple and pomegranate juice, pear sauce	(Patriarca et al., 2019; Pavicich et al., 2020; Taghizadeh et al., 2025; Vidal et al., 2019)
Ochratoxin A (OTA)	*Aspergillus carbonarius* *P. citrinum*	Apple and orange juice	(Carbonell-Rozas et al., 2024; Nan et al., 2022; Pallarés et al., 2019; Taghizadeh et al., 2025)
Aflatoxin B_1_ (AFB_1_)	*A. flavus* *A. parasiticus*	Sour cherry, orange, pineapple, peach, and guava juice	(Taghizadeh et al., 2022)
*Alternaria* *(*ALL)	*Alternaria alternata*	*Citrus, dried jujube, blueberry juice*	(Li et al., 2025; Pavicich et al., 2025)

### 5.2 Sulfur dioxide (SO_2_)

Control of wine spoilage is critical for winemakers to ensure the quality of their end product. The most employed solution against microbiological spoilage is the use of sulfite as the sulfur dioxide (SO_2)_ donor, which has exerted excellent antioxidant and antimicrobial properties, as well as increasing the solubility of polyphenols from fruit material in the winemaking. Inhibition of spoilage microorganisms is more challenging in fruit wine products with low alcohol content (5–10% v/v) and low acidity (above pH 3.7) than in the grape wines. In addition, it is a worldwide trend to reduce sulfite usage due to increasing public health concerns and tighter legislation regarding preservatives in drinks, considering their potential harmful effects such as DNA damage and respiratory disease. Recently, more and more natural plant extracts (plant polyphenols, polypeptides, and lysozyme) and physical treatments (UV irradiation, ultrasound, and electromagnetic and pulsed electronic fields) are attempted to replace SO_2_ during the winemaking (Feng et al., 2024). Nevertheless, some advantages have been demonstrated, it cannot be potentially completely replaced.

### 5.3 Biogenic amines and ethyl carbamate

Biogenic amines (BAs) are a class of organic base of low molecular weight derived internally from plants, fruits, and vegetables, it is proved that excessive intake of BAs can induce headache, gastralgia, severe palpitation, and respiratory distress. Up to now, approximately 20 types of BAs have been identified in fruit wine, but strict limitation of BAs in the fruit wines has not yet been established owing to less research on the BAs (Luo et al., 2024; Wang et al., 2026). BAs are produced via specific amino acid decarboxylation catalyzed by amino acid decarboxylases in microorganisms. Health supervision, selection of BA degradation strains, and optimization of processing conditions, as well as the use of natural extracts, could effectively inhibit decarboxylase activity and finally control the BA contents (Hong et al., 2025).

## 6 Conclusions and future perspectives

It is estimated that the global fruit wine market will keep a steady growth trend from 2025 to 2030, mainly driven by factors such as the healthy consumption concepts, product innovation, technological development, and policy support. The growth of the Chinese market is particularly significant, with the size exceeding 20 billion yuan in 2025 and reaching 100 billion yuan in the next five years, and female users accounting for over 68% (Zero Power, 2025).

### 6.1 Primary challenge

Aroma of fruit wine is a major determinant of its desirability by consumers, which mainly comes from three aspects. The first one refers to the specific aroma or compound aroma brought by the inherent aroma substances of different fruits, which is directly related to fruit varieties and harvest time, choice and use of enzyme, fruit crushing and antioxidation, juice extraction, as well as aroma protection and alcoholic fermentation. The second one refers to the fermented aroma substances brought by the microbial transformation of the components in the fruits; these substances usually include alcohols, esters, terpenes, etc. The selection of yeast strains and good management are critical. The last one is usually obtained by necessary technologies during aging, storage, and blending stages, supplementing complexity or uniqueness and harmony in the finished products. How to understand the typical aroma of different fruits and strike a balance between flavor and stability (especially color and colloidal stability) in the final products are primary challenges for fruit wine brewers. The technologies utilized in non-grape wine production were always drawn from the grape winemaking field, which easily leads to product homogenization to some extent. In practice, fruit wines often exhibit poor stability with a short shelf life when attempting to preserve the fruit's original flavor. Conversely, excessive focus on final product stability always led to significant flavor loss. This situation hinders the development of product standardization and robust market oversight. On the other hand, studies have emphasized the promising bioactive potential of fruit wines, but available literature still falls short compared to that for the fruit material itself. There remains a substantial gap in understanding fruit wines' effects on chronic human diseases such as cancer, cardiovascular disease, diabetes, neuro-degenerative diseases and more. Consequently, all the so-called health claims of numerous fruit wine products from markets is still disputed, necessitating further studies with animal models and/or human clinical trials are needed to verify their potential health benefits.

### 6.2 Future development trends

Non-grape fruit wine, as a diverse and highly promising fermented beverage, holds significant value in terms of flavor, nutrition, and economic benefits. Interdisciplinary fields such as multi-omics, modern microbial fermentation technology, and artificial intelligence play an important part in the development of non-grape fruit wine industry. A) Targeted regulation of fruit quality. Continuous development of precision plant breeding based on multi-omics technologies, including genomics and metabolomics, and gene editing technologies establishes a theoretical foundation for improving fruit quality through genome design. It has become feasible to accelerate the breeding process of superior fruit cultivars with multiple agronomic interests, good sensory, and other complex traits. B) Screening of fermentation strains. Targeted screening of native yeast respecting the fruit variety and regional flavor characteristics could enhance flavor complexity and express fruit flavors more accurately. Meantime, engineering strains based on genetic engineering breeding technology, precise fermentation and aging control technologies, and functional ingredient enhancement based on artificial intelligence will improve product quality and production efficiency of fruit wine. C) Innovation of brewing techniques‌. The production process is transforming from traditional alcohol fermentation of fruit juice to a combination of nutrition, functionality, and flavor, thereby enhancing aroma typicality and complexity and improving quality stability. In addition, reducing additive use deserves more attention. D) Novel product development. Complex fruit wine, low-alcohol fruit wine, and healthy fruit wine made of various fruits are future research hotspots. Combining tea, spices, honey, medicinal and edible plants, etc., the development of low-sugar, low-energy, and tonic fruit wine products will be the mainstream consumption trend in the future. E) Intelligence and sustainability. Artificial intelligence (AI) technology is increasingly employed in product and flavor innovation, fermentation process optimization, and brewing process control to advance sustainable development objectives. In summary, it should be focused on compensating for raw material defects for fruit winemaking, precisely managing fermentation, and balancing stability against sensory quality in future. Progress in this field will also depend on developing efficient sensory evaluation systems and establishing rigorous origin certification standards.

## CRediT authorship contribution statement

**Xiao Gong:** Writing – original draft, Project administration, Investigation, Funding acquisition, Data curation. **Ningli Qi:** Writing – review & editing, Resources, Conceptualization. **Biaoshi Wang:** Writing – review & editing, Methodology, Data curation, Conceptualization. **Tinghui Chen:** Writing – original draft, Resources, Formal analysis. **Yang Luo:** Writing – original draft, Software, Methodology, Funding acquisition. **Yiming Luo:** Validation, Methodology, Investigation, Formal analysis, Data curation.

## Ethical statement

This article is a review and does not involve any research with human participants or animal performed by an of the authors. All data and information discussed in this manuscript are derived from previously published literature, which has been appropriately cited.

The authors affirm that the manuscript complies with the ethical publishing standards and police of the target journals. No conflicts of interest or ethical issues are associated with the preparation of submission of this manuscript.

## Declaration of competing interest

The authors declare that they have no known competing financial interests or personal relationships that could have appeared to influence the work reported in this paper.

## Data Availability

Data will be made available on request.
